# Development of an Accurate Resonant Frequency Controlled Wire Ultrasound Surgical Instrument

**DOI:** 10.3390/s20113059

**Published:** 2020-05-28

**Authors:** Jungsuk Kim, Kyeongjin Kim, Sun-Ho Choe, Hojong Choi

**Affiliations:** 1 Department of Biomedical Engineering, Gachon University, 534-2, Hambakmoe-ro, Incheon 21936, Korea; jungsuk@bme.gachon.ac.kr; 2 Department of Medical IT Convergence Engineering, Kumoh National Institute of Technology, 350-27 Gumi-daero, Gumi 39253, Korea; 20196092@kumoh.ac.kr; 3 R&D Center, Metabiomed Corporation, 215 Osongsaenmyeong1-ro, Chenongu 28161, Korea; sognatore@metabiogw.bizmeka.com

**Keywords:** bolt-clamped Langevin ultrasonic transducer, wire ultrasound surgical instrument, generator instrument, handheld instrument

## Abstract

Our developed wire ultrasound surgical instrument comprises a bolt-clamped Langevin ultrasonic transducer (BLUT) fabricated by PMN-PZT single crystal material due to high mechanical quality factor and electromechanical coupling coefficient, a waveguide in the handheld instrument, and a generator instrument. To ensure high performance of wire ultrasound surgical instruments, the BLUT should vibrate at an accurate frequency because the BLUT’s frequency influences hemostasis and the effects of incisions on blood vessels and tissues. Therefore, we implemented a BLUT with a waveguide in the handheld instrument using a developed assembly jig process with impedance and network analyzers that can accurately control the compression force using a digital torque wrench. A generator instrument having a main control circuit with a low error rate, that is, an output frequency error rate within ±0.5% and an output voltage error rate within ±1.6%, was developed to generate the accurate frequency of the BLUT in the handheld instrument. In addition, a matching circuit between the BLUT and generator instrument with a network analyzer was developed to transfer displacement vibration efficiently from the handheld instrument to the end of the waveguide. Using the matching circuit, the measured S-parameter value of the generator instrument using a network analyzer was −24.3 dB at the resonant frequency. Thus, our proposed scheme can improve the vibration amplitude and accuracy of frequency control of the wire ultrasound surgical instrument due to developed PMN-PZT material and assembly jig process.

## 1. Introduction

Ultrasound has been used for a variety of applications, such as non-destructive testing, sound navigation, acoustic trapping, photoacoustic imaging, therapeutic high-intensity focused ultrasound devices, ultrasound cleaning, medical imaging, and surgical instruments [1,2,3,4,5,6,7,8,9,10]. A typical ultrasound instrument comprises an ultrasonic transducer with either only a transmitter or transceiver (transmitter and receiver) system [11,12]. In such an ultrasound instrument, the ultrasonic transducer is one of the most important devices [11,13,14,15]. This is because the ultrasonic transducer is itself a non-linear device composed of primary capacitive components having parasitic resistances, capacitances, and inductances in the equivalent circuit models [16,17,18]. Furthermore, the performance of the ultrasonic transducer is dependent on the driving voltages and frequencies of the transmitter or transceiver [19,20]. It is quite challenging to obtain stable performances from the ultrasonic transducers and other electronic instruments, irrespective of the frequency and voltage levels [6,21]. Therefore, the performances of the ultrasonic transducers could be sufficiently sensitive to affect the ultrasound performance of the entire instrument. Among the ultrasound instruments, the ultrasonic surgical instrument comprises a bolt-clamped Langevin ultrasonic transducer (BLUT) with a waveguide in the handheld instrument and a generator instrument [8]. The generator instrument used is a type of transmitter system that does not require a receiver system [21,22].

The ultrasonic surgical instrument is a surgical tool that is capable of hemostasis, coagulation, and incision of the soft or hard tissues using the frictional heat of a blade vibrated by the ultrasonic transducers [23]. It is applicable for use with soft and hard tissues depending on the areas of the human body [9]. In general, the generator instrument can control the output frequencies and amplitudes of the BLUT ultrasound transducer in the handheld instrument, following which ultrasonic energy amplification through the internal waveguide occurs, providing the hemostasis, coagulation, and incision capabilities using blade friction [9].

As the demand for minimally invasive surgery with fewer surgical scars and rapid patient recovery increases, the use of electro-surgery and ultrasound surgery machines continues to be on the rise [24]. Ultrasonic surgical machines are less damaged by heat at the incision area than electro-surgery machines, and they are cheaper and easier to operate than laser surgical machines [25]. The ultrasound surgical instruments used for soft tissues are mainly manufactured by Ethicon, Olympus, and Covidien Corp. In 2013, Covidien provided the wireless ultrasound surgical instrument (product name: Sonicision) to provide freedom of movement because of wire reduction [26]. In 2015, Ethicon Corp. manufactures the wire ultrasound surgical instrument (product name: Ethicon) to carry out hemostasis on blood vessels up to 7 mm in length [24]. In 2016, Lotus supplied the wire ultrasound surgery scalpel to generate torsional rotation instead of linear motion in order to increase the power efficiency [25]. In 2017, Olympus Corp. developed the wire electrical/ultrasound surgical instruments (product name: Thunderbeat) to reduce the incision operation time by using the combined power of two electrical/ultrasound sources [27]. Currently, the technology demands of the ultrasound surgical instruments for soft tissues are for the improvement of the coagulation and incision of the soft tissues and effective residual heat treatment after incision [26,28]. Therefore, accurate frequency control of the main device, which is the BLUT, could affect the aggregate performance of the wire ultrasound surgical instrument.

Currently used ultrasound surgical instruments use PZT single polycrystalline materials, but we used PMN-PZT single crystal material for ultrasound surgical instruments [3,17,29,30,31]. Since the mechanical quality factor and electromechanical coupling coefficient of the PMN-PZT material are higher than those of PZT material, the output power of the PMN-PZT material is higher than that of the PZT material [3,32,33]. Compared to PZT material, PMN-PZT material could make a smaller piezoelectric transducer which could possibly reduce unnecessary lateral vibrations inside the handpiece if the same output power is used. Therefore, the developed instrument could possibly improve the incision effects on the blood vessels and tissues.

In addition, a novel assembly process was developed using a jig station with impedance and network analyzers to produce accurate frequency control of the handpiece component and instrument. This process can reduce vertical vibration loss and generate alignment adjustment and maintenance when assembling the BLUT and the metal blocks very accurately. In addition, this developed jig station combined with impedance analyzer can provide mounting and desorption of the BLUT easily, so it can apply preload to the piezoelectric body and metal block accurately. To minimize the vibration loss of the BLUT, the resonant frequency of the handpiece and hand instrument need to be matched. Therefore, a matching circuit was developed to minimize the return loss between the handpiece and hand instrument using the network analyzer. As a result of that, we can expect that vibration amplitude and frequency error of proposed instrument are more enhanced than currently used ultrasound surgical instrument.

Section 2 describes the design of the BLUT, waveguide, and the handheld instrument. Section 3 discusses the manufacture of the BLUT via the use of a digital torque wrench and explains the development of the generator instrument. Furthermore, the analysis and performance measurements of the BLUT in combination with a waveguide, and those of the generator instrument are described. Section 4 discusses the conclusions of the research study.

## 2. Materials and Methods

In the case of the BLUT, the resonance frequency in the primary longitudinal vibration mode is utilized to obtain the maximum longitudinal vibration using the wavelength in the material, as shown in Equation (1) [1,34]. The formula to find the length of the piezoelectric material or medium for the BLUT (*L*) is as shown below [19]. Therefore, the length of the piezoelectric material can be obtained as stated below.

(1) $$\lambda =\frac{c}{f},L=\frac{c}{2f},\mathrm{and}L=\frac{\lambda }{2},$$

where *λ*, *c*, and *f* are the ultrasound wavelength, velocity, and frequency of the piezoelectric materials, respectively.

This equation dictates that the piezoelectric material can be resonated if the *L* value is equal to half of the wavelength. The BLUT consists of a mass of a certain type of material in the front portion of its piezoelectric body, and a mass of a different type of material at its back. Therefore, it must be designed after factoring in the parameters of its different compositional materials, as shown in Equation (2) [3,35].

(2) $$k=\frac{c_{1}\rho_{1}s_{1}}{c_{2}\rho_{2}s_{2}}=\frac{Z_{m}}{Z_{p}},$$

where *c*_1_ and *c*_2_, *ρ*_1_, and *ρ*_2_, and *s*_1_ and *s*_2_ are the velocity, density, and cross-sectional areas of the piezoelectric and metal contact surfaces, respectively; and *Z*_p_ and *Z*_m_ are the mechanical impedances of the piezoelectric and metallic contact surfaces, respectively.

**Figure 1:**
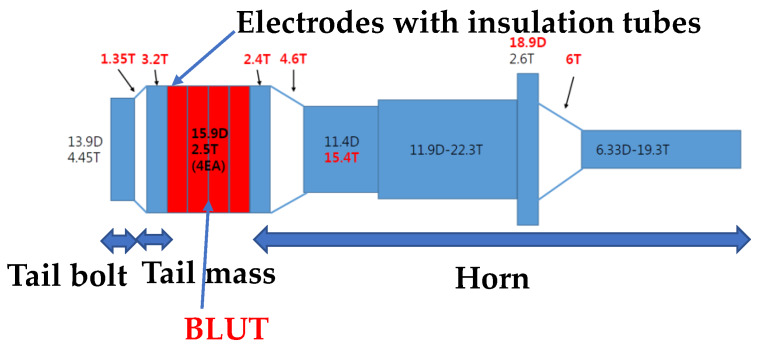
Shape of BLUT.

The measured quality factor of the PMN-PZT (1000) is also higher than that of PZT-4 polycrystalline material (500). The mechanical electrical coupling coefficient of the PMN-PZT (0.91) is also higher than that of PZT-4 polycrystalline material (0.7). For the same volume, the measured output power of the PMN-PZT (60 W/cm^3^) is higher than that of the PZT-4 polycrystalline material (41 W/cm^3^). Based on the design formulas above, the target specifications, selected materials, and the basic shapes of the BLUT with a handset were designed as shown in Figure 1. The parts of the device colored red and those colored blue and white represent the piezoelectric material and the hand-piece, respectively. The diameter of each metal part was designed based on the piezoelectric body diameter, and the diameter of the horn part was designed based on the amplification ratio. The wavelength was calculated based on the quantitative target frequency and the length of the horn and tail mass, and the tail bolt was designed based on longitudinal velocity. Since the piezoelectric material has anisotropy by the piezoelectric polarization effect, the harmonic analysis is applied to obtain the quantitative values, as shown in Results and Discussion. “T” and “D” denote the diameter and length and their units are mm.

**Figure 2:**
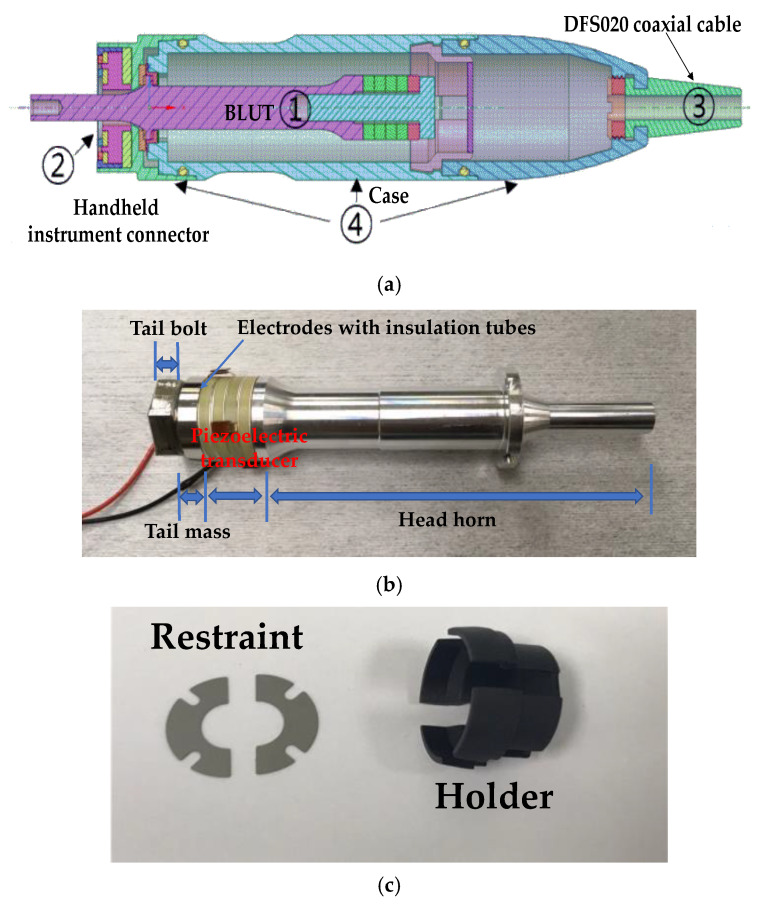
(**a**) Modeled handpiece. (**b**) Handpiece with BLUT. (**c**) Restraint and holder components.

The entire wire ultrasound surgical instrument that we developed is composed of a hand-piece, a handle, a waveguide in the handheld instrument, and the generator instrument. As shown in Figure 2a, the hand-piece consists of the BLUT (①) in combination with a BLUT restraint, and the handheld instrument connector (②), the connecting cable (③), and parts comprising the case (④). As shown in Figure 2b, the BLUT consists of the head horn, piezoelectric transducer element, tail mass, tail bolt, and electrodes with insulation tubes. As shown in Figure 2c, the BLUT restraint was designed such that it would not induce interference during the transmission of vibration. The front fixing plate was used to prevent detachment by placing the transducer on the case. The holder was designed so that the rear section of the transducer could be attached to permit the optimal placement of both the wire and the minimum/maximum (Min/Max) button that controls the driving signal. This allowed the signal and ground area to be separately connected to instrument. A 50-Ω DFS020 coaxial cable with a single wire serving the function of signal/ground wires for electrical impedance matching was used. This cable was used to withstand at least 150V_p-p_ and 150 °C. The output jacket was made of silicone for flexibility, insulation, and chemical resistance.

**Figure 3:**
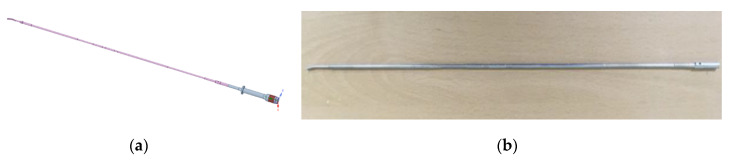
(**a**) A model 36 cm waveguide in the handheld instrument; (**b**) a fabricated 36 cm waveguide.

The BLUT has to be designed to produce maximum displacement at the end of the waveguide. Therefore, the length of the waveguide is supposed to be half of the wavelength of the piezoelectric materials. The selected piezoelectric material is required to possess excellent mechanical qualities and a high electromechanical coupling coefficient [17]. The waveguide component is used to perform the surgical incision, and therefore, it is made of Ti-6Al-4V ELI titanium alloy that exhibits outstanding strength and biocompatibility [36]. The waveguide part is composed of components such as a jaw combined with an inner-outer pipe, a pin, a waveguide fastening component, and a knob. The waveguide component for transmitting the generated vibration output from the BLUT into the surgical target had to be designed. As shown in Figure 3, a 36 cm laparoscopic waveguide with a hand-piece was designed for the single crystal BLUT. The pinholes present in the handheld instrument tube were placed at the ½ *λ* position, specifically, at the point where the longitudinal vibration amplitude converged to zero. Furthermore, the portion of the transducer that reached the maximum vibration amplitude was placed at ¼ *λ*, as confirmed in the results.

**Figure 4:**
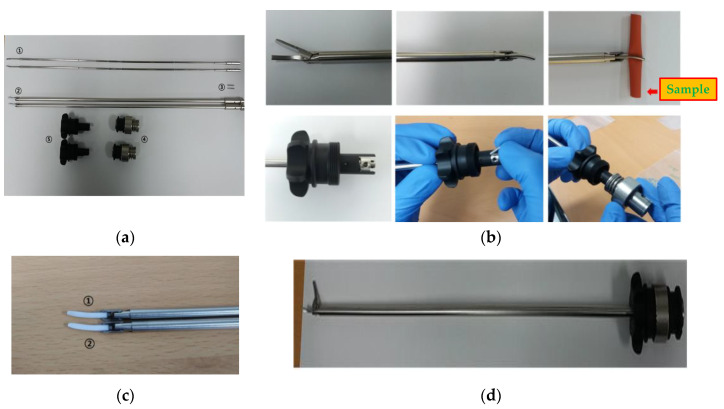
(**a**) Waveguide components; (**b**) assembly of waveguide component; (**c**) jaw pad; (**d**) prototype of the waveguide.

The waveguide of the ultrasonic surgery device makes contact with living tissue, and therefore, it is preferable that no hint of magnetism is evident during the surgery. STS304, which is the material used to manufacture the pipe of the waveguide component, is not magnetic. However, some material may become magnetized when the size of the waveguide is adjusted. Therefore, the primary magnet was removed through the annealing processes and the secondary magnet was removed using the demagnetizer, in order to improve the performance of the waveguide. In Figure 4a, the waveguide is composed of the waveguide itself (①), the jaw and the inner-outer pipe (②), the pin (③), waveguide fastening apparatus (④), and the components of the knob (⑤). As shown in Figure 4b, the waveguide was assembled by connecting the waveguide, jaw and inner-outer pipe, and the knob, with pins. The sample needs to be fixed between the braid and the jaw sections of the waveguide, so that the curvature of the braid and jaw section can be matched. As shown in Figure 4c, the jaw pad grips were manufactured in thin (①) and thick (②) forms to ensure tight contact between the waveguide and sample. It was confirmed that the grip of the jaw pad was in closer contact with the smaller sized samples of tissue in the case of the thin form rather than with the thick form; therefore, the final shape of the jaw pad was selected to be the thin grip. The final assembled prototype of waveguide is as shown in Figure 4d.

**Figure 5:**
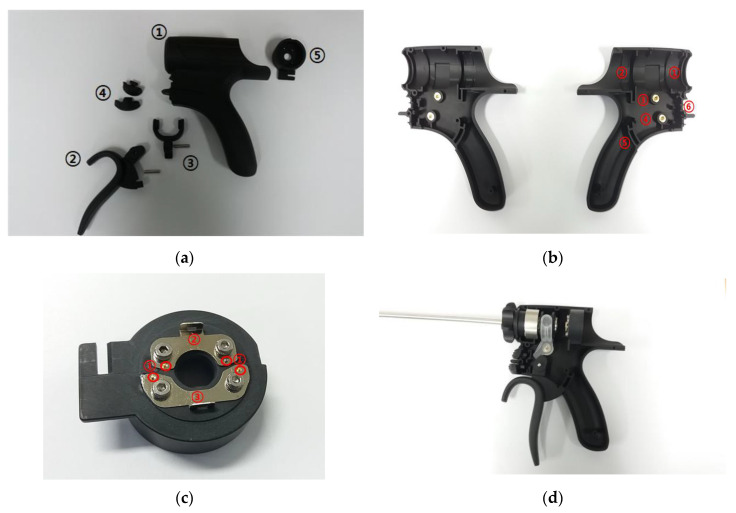
(**a**) Separate parts of the handheld instrument in its case. (**b**) Handle of the handheld instrument. (**c**) Terminal section. (**d**) Assembled handheld instrument in its case.

As shown in Figure 5a, the entire handheld instrument consists of the body (①), the handle (②), the waveguide fixing ring (③), Min/Max switch (④), and terminal (⑤). As shown in Figure 5b, the body part consists of the waveguide fixing component (①), the hand-piece fixing component (②), the fixing ring pin component for the waveguide (③), the handle fixing pin (④), the handle guide (⑤), and the Min/Max switch fixing component (⑥). By reducing the length of the handle guide, the design of the handle facilitates easy operation. The terminal consists of pogo pin holes (①) and terminal plates (②), as shown in Figure 5c. Figure 5d shows the assembled waveguide component in the handheld instrument.

**Figure 6:**
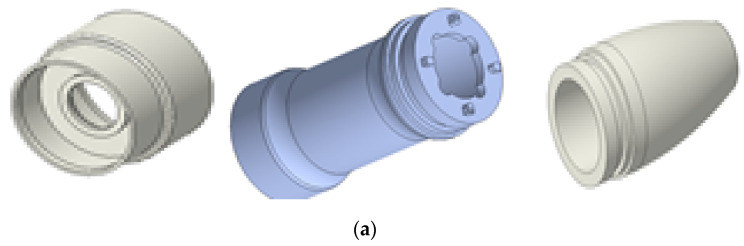

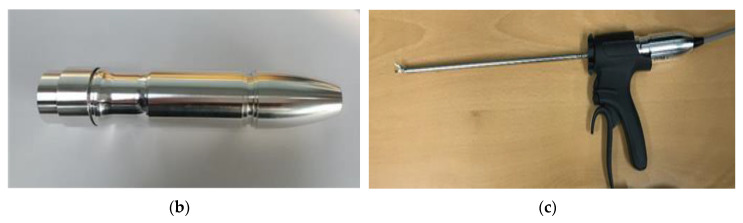
(**a**) 3D model and (**b**) assembled outer case part. (**c**) Assembled BLUT, waveguide, and handheld instrument.

Figure 6a,b displays the 3D model and the assembled prototype of the outer case component for the hand-piece. The case is composed of three components used in BLUTs and cables; therefore, the aluminum cases were manufactured to optimize durability and weight. Figure 6c shows the 36 cm waveguide and the handheld instrument.

**Figure 7:**
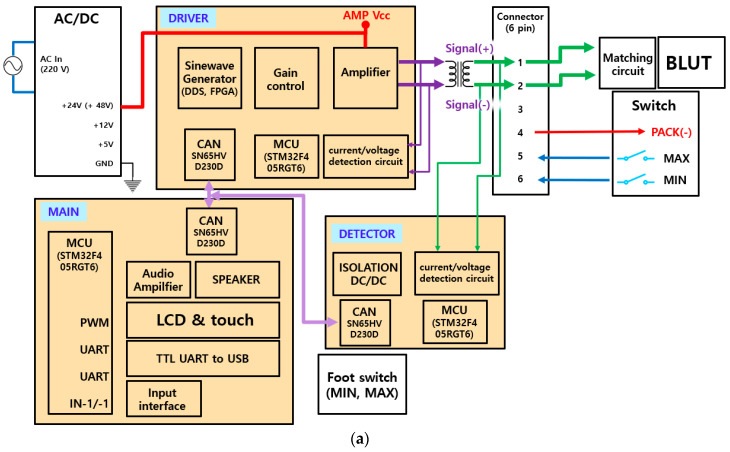

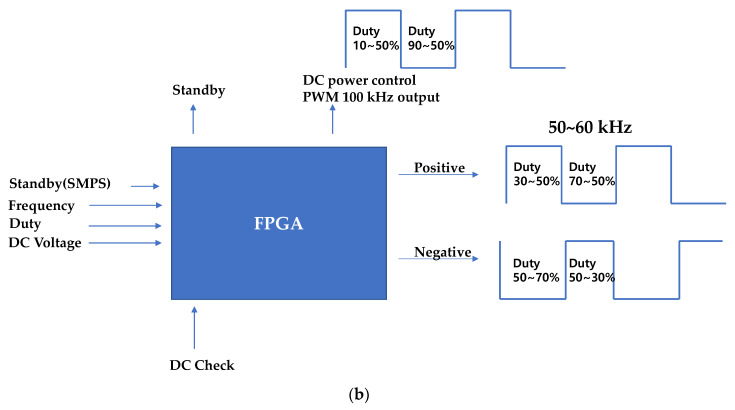
Block diagrams of (**a**) generator configuration and (**b**) PWM signal generation from the FPGA board.

The block diagram of the generator instrument including the matching circuit and the BLUT is displayed in Figure 7a. The generator instrument is composed of a 100 W variable switching mode power supply (SMPS) circuit, a control circuit, an ultrasonic driving circuit, a matching circuit, and an output monitoring circuit. The input voltage (AC In) is 220 V at a frequency of 50–60 Hz. The target output frequency of the generator is 55.5 kHz with a ±3% error rate. The generator circuits (DRIVER) were used to generate the sine waveform from the pulse width modulation (PWM) signal. The main control circuits (MAIN) control the generator circuits and monitoring circuits (DETECTOR). The main control circuit receives the external inputs and controls the ultrasonic outputs through the output control circuit. Further, it displays the voltages and currents in the external display using serial communication. Through direct digital synthesis (DDS) and field-programmable gate arrays (FPGA), sine waveforms were generated and were amplified in several stages. These sine waves were also transformer controlled using external monitoring circuits (DETECTOR) in addition to a matching circuit and BLUTs (BLUT). Meanwhile, the generator input/output waveforms were detected by employing current/voltage detection circuits. Subsequently, they were applied in algorithms deployed in the micro controller unit (MCU) to adjust the amplification rate of the output voltage and current according to frequency. As shown in Figure 7b, the variable SMPS circuit generates voltages of magnitude 30–150 V. Therefore, it can be used to control output voltages in the amplifier circuit. The user-defined frequency was generated to control the duty cycle.

## 3. Results and Discussion

**Figure 8:**
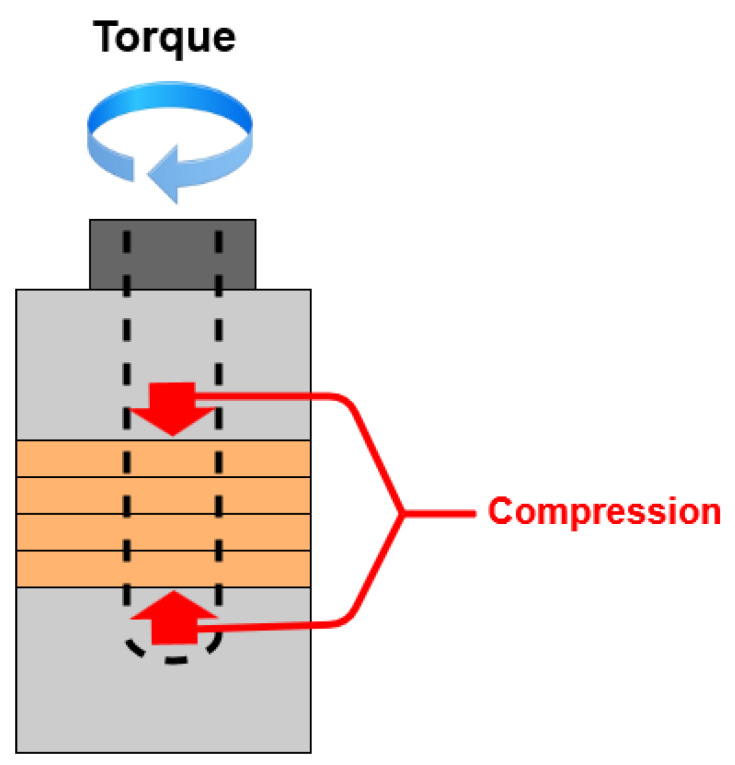
Compression forces according to the torque fluctuation.

Figure 8 displays the compression forces when a BLUT is subjected to torque fluctuations. In the BLUT, the piezoelectric element is pre-loaded by the compression force caused by the bolt-tightening torque. Therefore, the vibration characteristics, including the maximum vibration amplitude and resonance frequencies, change according to variations in the pre-pressure. When excessive stress is generated in the piezoelectric element because of the pre-pressure, it is recommended that a single crystal piezoelectric element can be used at a stress of about 40 MPa or less. This is because de-polarization occurs, wherein the arrangement of the internal dipole moment is distributed [37]. The formula for the screwing force relation can be used to estimate the compressive forces in the absence and presence of the tightening torques. Therefore, Equations (3) and (4) represent the clamping force (*Q*) and stress (*σ*), respectively [38,39].

(3) $$Q=\frac{T}{\frac{d_{2}}{2}\tan\left(\tan^{-1}\left(\frac{\mu }{\cos\frac{\alpha }{2}}\right)+\beta \right)},$$

(4) $$\sigma =\frac{F}{A},$$

where *T* is the tightening torque (N·m), *µ* is the friction factor, *d_2_* is the bolt effective diameter, *α* is the screw thread angle, *β* is the screw lead angle, *σ* is the stress (MPa), *F* is the weight (N), and *A* is the cross-section area (m^2^).

**Figure 9:**
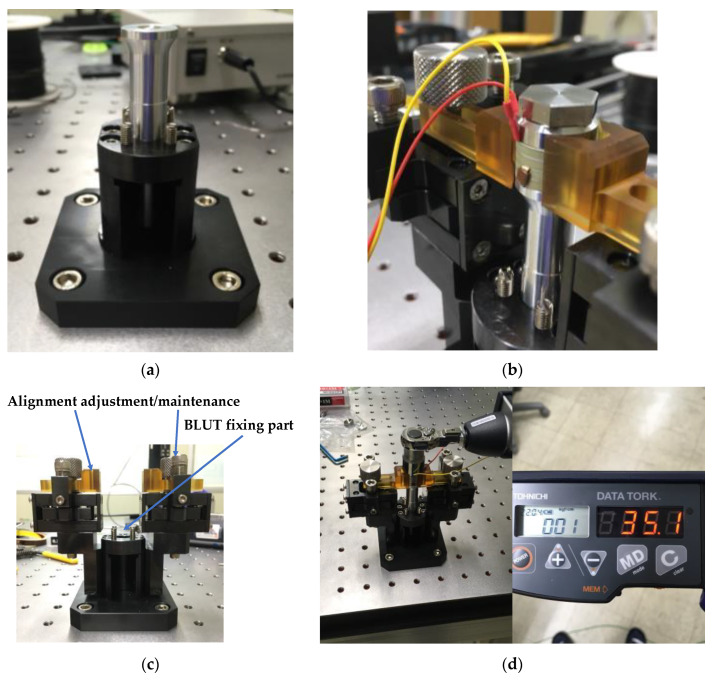
(**a**) Front mass fixing and (**b**) alignment adjustment/maintenance parts; (**c**) assembly jig station; (**d**) manufactured parts for BLUT with digital torque wrench.

In order to assemble and control the BLUT accurately, a new assembly jig process using impedance and network analyzers was developed for the purpose of accurate bolting torque management, alignment adjustment, and ease of maintenance between the piezoelectric element and the metal block parts, thereby improving the frequency control accuracy of the piezoelectric transducers. Figure 9a–d depict the mass fixing component at the front, the alignment adjustment/maintenance sections, the assembly jig, and the manufactured components of the BLUT in combination with a digital torque wrench, respectively. We used a commercial torque wrench in our proposed process. To reduce the short circuit between the source and ground of the piezoelectric material, the alignment adjustment/maintenance components were insulated by employing Ultem material, which is amorphous, possesses high mechanical strength and rigidity, and was developed by General Electric USA [40]. Thus, there is no short-circuit between the two piezoelectric materials that might lead to an aggregation of their powers owing to the compressive force experienced during the bolting process. As shown in Figure 9c, there are the part for adjusting the alignment and maintenance of the BLUT and fixing BLUT. This process can reduce vertical vibration loss and generate alignment adjustment and maintenance when assembling the BLUT and the metal blocks. In addition, it can apply preload to the piezoelectric body and metal block accurately. As shown in Figure 9d, the manufactured parts for the BLUT were assembled and fabricated using an assembly jig and by employing a commercial digital torque wrench. The BLUT was manufactured by presetting the working torque in the digital torque wrench in kgf cm units because the compressive force experienced by the piezoelectric elements varies. Furthermore, the resonance frequency and output characteristics of the piezoelectric elements also change according to the fluctuations in the bolt-tightening torque [38].

However, it may not be accurate enough to calculate the compressive forces for the BLUT because some measured parameters might not be accurate under tightening torques [38]. Therefore, we further performed the actual measurement using the assembly jig station and the impedance analyzer. The impedance characteristics of the BLUT sensitive to the compressive stress were checked and reflected during manufacturing process. To verify the resonance frequency changes according to the bolting torque, the torque was initially increased in 10kgf ·cm increments. Following this step, the resonance frequency was measured using an impedance analyzer (E4194A, Agilent Technologies, Santa Clara, CA, USA). The actual ultrasonic surgical instrument was operated in combination with the waveguide in the handheld instrument. Therefore, we compared the resonance frequencies obtained with and without the waveguide. This step is necessary, because the impedance of the ultrasonic transducer could be modified by the use of an additional component [18].

**Figure 10:**
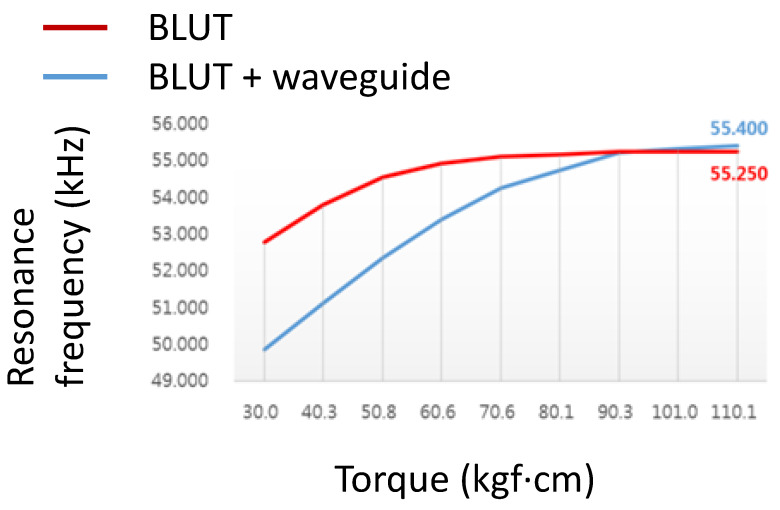
Resonance frequency of the BLUT with and without waveguide.

Figure 10 shows the resonance frequency graph of the BLUT with and without the waveguide in the handheld instrument. The resonant frequencies were obtained using the impedance analyzer (E4194A). The first resonant frequency of the BLUT with and without the waveguide was evident at a torque of 10 kgf·cm. In the case of the BLUT, the rise in resonant frequency was steep up to a torque of 90.3 kgf·cm. After that point, it only increased slightly with higher torques. However, in the case of the BLUT that employed a waveguide, the resonant frequency rise was steep up to a torque of 50.08 kgf·cm. Therefore, the rise in the frequency of the BLUT-waveguide combination was much less than that of the stand-alone BLUT. Furthermore, the frequency rise exhibited saturation at a resonance frequency of 55.25 kHz at a torque of 90.3 kgf·cm. Using such experimental data, the maximum working torque was set to about 100 kgf·cm. This result confirms that the resonant frequency can be varied within a certain range.

**Figure 11:**
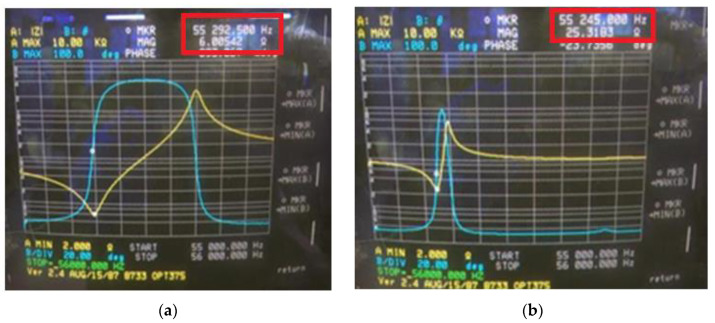
Measured electrical impedance versus frequency of the BLUT (**a**) with and (**b**) without waveguide.

With a tightening torque of about 100 kgf·cm, the BLUT with waveguide was fabricated to verify the resonance frequencies and electrical impedances. Figure 11 shows the measured electrical impedance versus the frequency of the BLUT with and without a waveguide in the impedance analyzer (E4941A). The resonant frequency and the electrical impedance of the BLUT by itself were 55.29 kHz and 6.0 Ω, respectively. The resonant frequency and electrical impedance of the BLUT with waveguide were 55.24 kHz and 25.31 Ω, respectively. Furthermore, the matching conditions between developed handpiece with waveguide and instrument were verified using network analyzer to minimize the signal loss because the accurate frequency control is also affected when connecting the handpiece with waveguide and the instrument.

**Figure 12:**
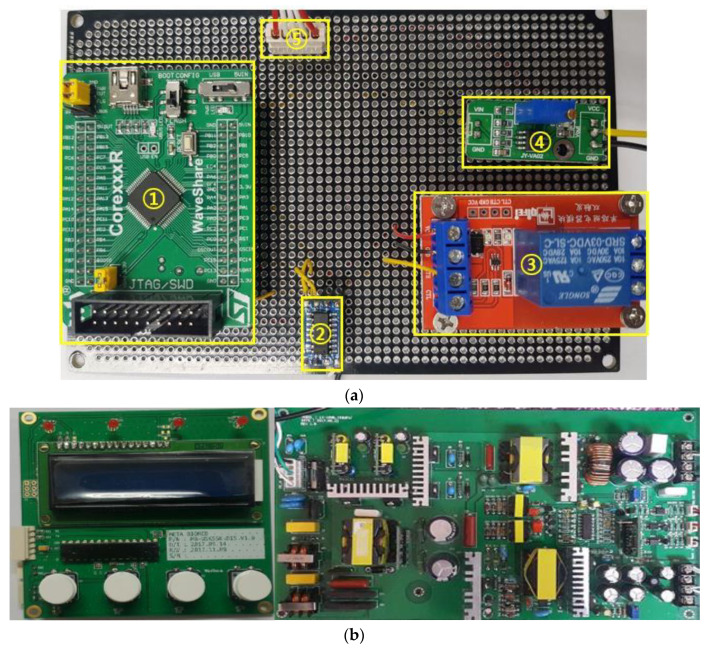
(**a**) Fabricated generator PCB board with minimum/maximum switch input control; (**b**) fabricated display PCB and switching mode power supply (SMPS) circuits.

As shown in Figure 12a, in the generator instrument, the first section comprises (①) the MCU module to control the universal asynchronous receiver/transmitter (UART), the minimum/maximum (Min/Max) foot switch, the analog-to-digital converter (ADC) input, the digital-to-analog converter (DAC) output, and the on/off output relay control to communication with the LCD. The second section (②) consists of the UART interface module for Min/Max and touch control, and the level communication of LCD modules. The third section (③) is the relay module to control actual on/off output when the Min/Max switch input is generated. The fourth section (④) is the operational amplifier (OPAMP) to boost the DAC output level from a range of 0–3.3 V to 0–5 V. Figure 12b displays the printed circuit board (PCB) component and the SMPS circuit. The fifth section (⑤) is the module power supply of 12 V and 5 V and switch ADC input cable connectors. In the LCD display, the current output voltage and output frequency are displayed (e.g., 50V DC or 60VDC with 55.3 kHz).

In the generator instrument, the main control circuit is important because it receives the external input and controls the ultrasonic output signals through the output control circuit. Furthermore, it displays the voltages and currents in the external display. Therefore, the measured output frequencies and output amplitudes of the main control circuit in the generator instrument are shown in Table 1 and Table 2. The output frequency of the main control circuit was measured as the input frequency was varied. The output voltage was set to 60 V. The input frequency was changed from 50 to 60 kHz in 1 kHz increments, and subsequently, the output frequencies were measured using a frequency counter. From the measurements (Table 1), the output frequency error rate was within ±0.5%, which demonstrates an accurate control of the output frequency of the main control circuit.

The measured output voltage amplitudes of the main control circuit in the generator instrument are shown in Table 2. The output voltages of the main control circuit were measured. The output frequency was set to 55.5 kHz. The input voltages were changed from 30 to 150 V in 10 V increments, following which step, the output voltages using an oscilloscope were measured. Because of the measurement (Table 2), the output voltage error rate was within ±1.6%, which implies an accurate output control voltage in the main control circuit.

**Figure 13:**
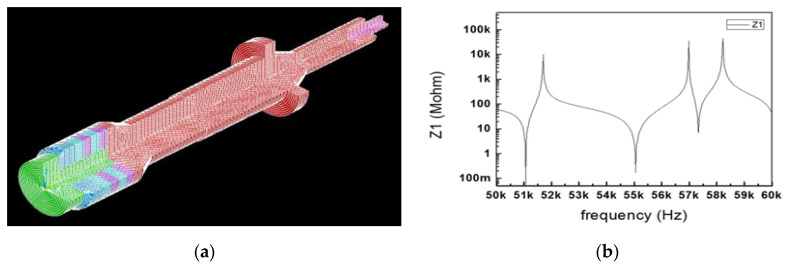

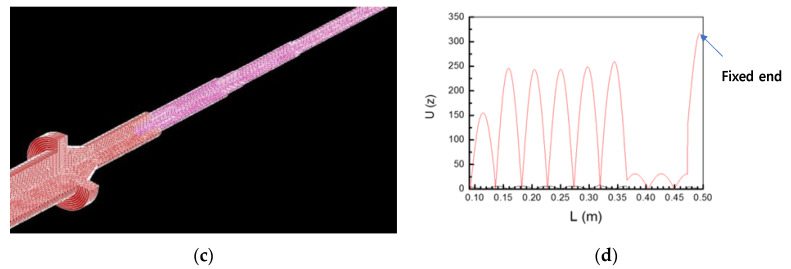
(**a**) Model analysis of the BLUT. (**b**) Impedance vs. frequency graph of the BLUT. (**c**) displacement model and (**d**) data of BLUT combined with the waveguide 3D assembly model.

In Figure 13a, modal analysis was performed to confirm that the longitudinal natural frequency of the BLUT fabricated by PMN-PZT material is close to the target frequencies, as shown in Figure 11a. In the modal analysis, the longitudinal natural frequency was 55.21 kHz, as shown in Figure 13b.

Harmonic analysis is used to calculate the vibrational displacement or stress at a structural node if the constant waveform is excited [38]. As shown in Figure 13c, the maximum longitudinal vibration displacement was obtained at the blade ends of the BLUT fabricated by PMN-PZT material when combined with waveguide assembly models in the harmonic response analysis. The longitudinal vibration displacement data at the coordinates of each node were extracted from the harmonic response analysis. In Figure 13d, the X and Y-axes represent the length and the values of the longitudinal displacement of the BLUT-waveguide combination. The maximum longitudinal displacement value was 317 µm at 55.06 kHz, as shown at the fixed end point. The lowest longitudinal displacement point is near the fixed end of the BLUT. Therefore, the displacement converges to zero under fixed conditions.

**Figure 14:**
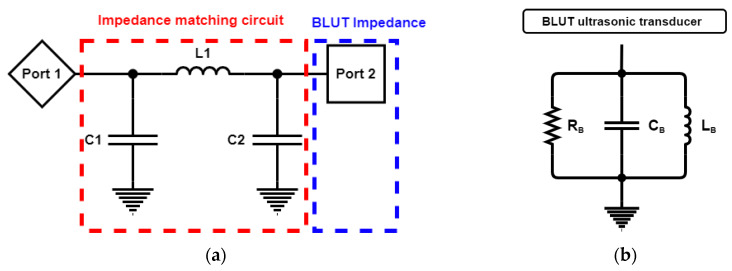

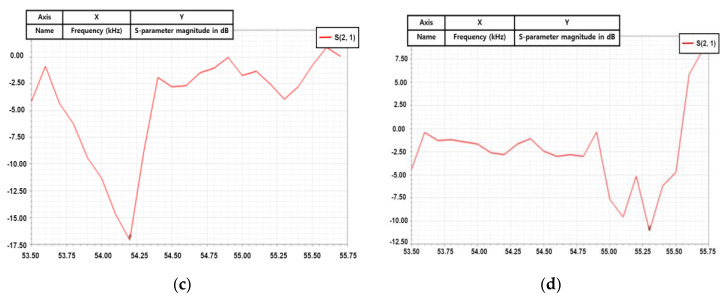
(**a**) Simulation model of the impedance matching circuit with the BLUT. (**b**) Equivalent circuit model of BLUT. S-parameter values vs. frequency (**c**) without a matching circuit and (**d**) with matching circuit.

Figure 14a depicts the impedance matching circuit between the generator instrument (Port 1) and the BLUT (Port 2) that was designed to reduce reflection loss [41]. Figure 14b shows the measured equivalent circuit model of the BLUT using an impedance analyzer (E4194A, Agilent Technologies, Santa Clara, CA, USA). The resistance, capacitance, and inductance values of the measured equivalent circuit model are 14.51 kΩ, 281.396 nF, and 28.1841 µH, respectively. Using the S-parameter analysis in the ADS circuit simulation program (Agilent Technology, Santa Clara, CA, USA), matching circuit values could be estimated as shown in Figure 14c [42]. The S-parameter analysis was used to maximize the power transfer from the generator instrument to the BLUT. It is usually desirable to have a S-parameter value that is less than −10 dB in electronic component designs [5,43,44,45]. The resonant frequency of the generator that includes the matching circuit and the BLUT needs to be around 55 kHz. Before using the matching circuit, the generator with the BLUT has a resonant frequency of 54.2 kHz, as shown in Figure 14c. After using the matching circuit, the generator with the BLUT has its resonant frequency changed to 55.3 kHz, as shown in Figure 14d. After obtaining the simulation results, the values of the capacitances (C_1_ and C_2_) and inductance are determined as 1.5 nF (C_1_), 7.2 nF (C_2_), and 330 µH (L_1_).

Using a network analyzer, the measured S-parameter performance of the generator with and without the matching circuit was obtained in order to verify the effective power transfer [46,47]. The matching circuit values through simulation were applied to the generator instrument, following which step, performances were measured. This step was carried out because simulated and measured performances under a high-voltage environment are not accurate [48,49,50]. Without the matching circuit, the value of the input S-parameter of the generator is −5.7 dB at the resonant frequency, which indicates that the source of the driving signal is more attributable to the BLUT. This could result in a deterioration of the performance metrics of the transducer, such as in the bandwidths and amplitudes of the echo signals. With a matching circuit, the value of the input S-parameter of the generator is −24.3 dB at the resonant frequency, which is well matched at the resonant frequency of the transducer.

**Figure 15:**
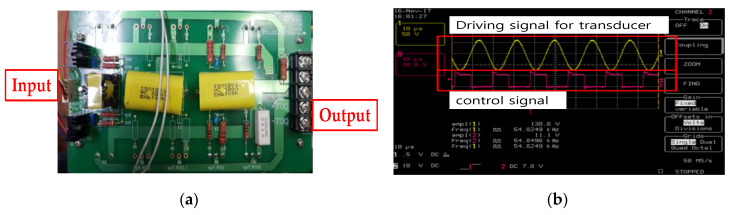
(**a**) Fabricated driving circuit and (**b**) driving and control signal for the BLUT.

To generate ultrasonic driving signals, a typical push-pull type amplifier was used so that positive and negative PWM signals were generated at a controlled frequency and voltage, as shown in Figure 15a. In Figure 15b, the external input frequencies and voltages were set, following which, output frequencies and voltages were tested using an oscilloscope. The output frequency and voltage of driving signal were 54.82 kHz and 130 V_p-p_.

**Figure 16:**
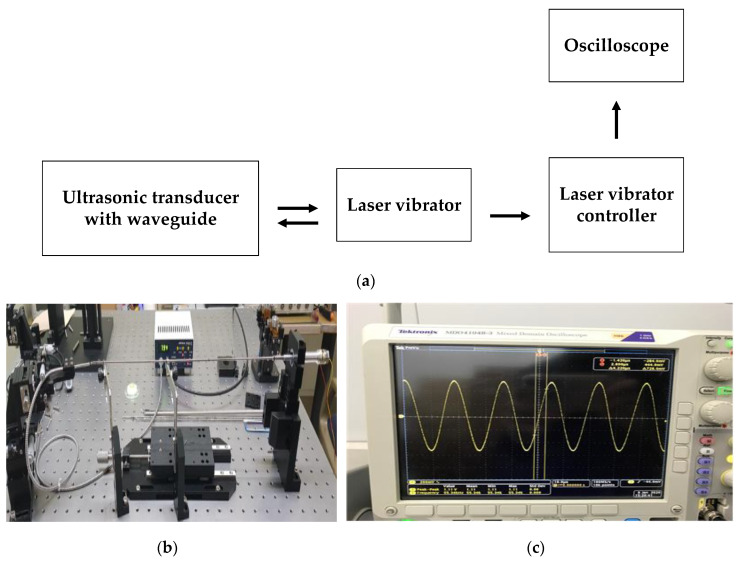
(**a**) Setup diagram and (**b**) picture to measure the amplitude and vibration of the BLUTs; (**c**) measured voltage signal in the oscilloscope.

The maximum target vibration frequencies and amplitudes of the blade-end component in the BLUT-waveguide that we developed are 55.5 kHz with ±3% error rates and 100 μm. The maximum vibration amplitudes and frequencies of the developed BLUT in the handheld instrument were measured using a displacement measurement meter connected to an oscilloscope, as shown in Figure 16a. As shown in the measurement setup (Figure 16b), the laser vibrator under the control of the laser vibrator controller (PM-E, Unipulse Corp. Toyoko, Japan) transmitted the light to the BLUT that we developed, which is placed in the handheld instrument. Subsequently, the returned light was detected by the laser vibrator. The vibrator controller received the vibration to be converted into the voltage displayed in the oscilloscope as depicted in Figure 16c. The displacement measured by the optical vibrator equipment was displayed as a voltage in the oscilloscope. Thus, the voltages have to be converted into a linear form. The maximum longitudinal vibration displacement voltage was measured as 27.3 mV. The actual displacement amplitude can be calculated by multiplying the voltage with the corresponding linear displacement of 250.4 um/V in the laser vibrator equipment. Therefore, the measured minimum and maximum vibration amplitudes were 36.7 and 99.4 μm, which are higher than those of the commercial instrument (30 μm). From this result, we expect that the vibration amplitude in the blade-end of the BLUT-waveguide combination would be improved. The minimum and maximum vibration frequencies of the BLUT waveguide combination were 55.34 and 55.5 kHz. Therefore, the measured vibration frequency error rate was 0.28%, which is much less than that of the commercial instrument (±5%).

## 4. Conclusions

Wire ultrasound surgical instruments are useful surgical tool for hemostasis, coagulation, and in the incision of tissues via the use of the vibrations from the ultrasonic transducer. It is very important to maintain the accurate frequency of the BLUT for the performances of the wire ultrasound surgical instruments because the frequency of the transducer is related to the hemostasis procedure and the effects of the incision on the blood vessels and tissue. Therefore, the BLUT fabricated by newly developed PMN-PZT single crystal material due to high mechanical quality factor and electromechanical coupling coefficient was designed to improve the frequency accuracy of the wire ultrasound surgical instrument. For same volume, the measured output power of the PMN-PZT is higher than that of the PZT-4 polycrystalline material. In addition, the mechanical coupling coefficient and quality factor of the PMN-PZT are also higher than those of the PZT-4 polycrystalline material.

The developed assembly jig process using impedance and network analyzers can control the compression force accurately by using the torque fluctuation in the case of the BLUT–waveguide combination and instrument. The resonant frequencies that depended on the magnitude of the torque were measured to obtain the stable performance metrics of the BLUT–waveguide. This developed jig station combined with impedance analyzer can provide mounting and desorption of the BLUT easily to minimize the vibration loss of the BLUT. To reduce the short circuit between the source and ground of the piezoelectric material, the alignment adjustment/maintenance components were insulated by employing Ultem material.

To support the BLUT that we developed, a handheld instrument was also developed with a main control circuit, whose output frequency error rate was within ±0.5% and whose output voltage error rate was within ±1.6%. In addition, an impedance matching circuit was developed to maximize the power transfer at the resonant frequency between the handheld instrument and the BLUT. With a matching circuit, the S-parameter value is −24.3 dB at the resonant frequency in the network analyzer. The error rate of the measured vibration frequency of BLUT is 0.28%. As a result of that, the vibration amplitude and frequency error of the proposed instrument are more enhanced than in the commercial product. Therefore, we conclude that this wire ultrasound surgical instrument could lead to improvements in the hemostasis procedure and ameliorate the effects of incision on blood vessels and tissues.

## Figures and Tables

**Table 1:** Measured input and output frequencies of the main control circuit with error rates.

Input Frequency (kHz)	Output Frequency (kHz)	Error Rate (%)
50	49.95	−0.20
51	50.97	−0.04
52	51.98	−0.15
53	52.97	−0.17
54	54.00	−0.09
55	54.95	−0.09
56	55.99	−0.32
57	56.95	−0.05
58	57.94	+0.34
59	58.96	−0.44
60	59.95	+0.20

**Table 2:** Measured input and output amplitudes of the main control circuit with error rates.

Input Voltage (V)	Output Voltage (V)	Error Rate (%)
30	29.52	+1.60
40	39.66	+0.85
50	49.89	+0.22
60	59.97	+0.05
70	70.02	−0.03
80	80.03	−0.04
90	90.22	−0.24
100	100.5	−0.50
110	110.8	−0.73
120	121.1	−0.92
130	131.5	−1.15
140	141.8	−1.29
150	150.6	−0.40
